# The genome of *Rhizophagus clarus* HR1 reveals a common genetic basis for auxotrophy among arbuscular mycorrhizal fungi

**DOI:** 10.1186/s12864-018-4853-0

**Published:** 2018-06-18

**Authors:** Yuuki Kobayashi, Taro Maeda, Katsushi Yamaguchi, Hiromu Kameoka, Sachiko Tanaka, Tatsuhiro Ezawa, Shuji Shigenobu, Masayoshi Kawaguchi

**Affiliations:** 1 Division of Symbiotic Systems, National Institute for Basic Biology, Okazaki, Japan; 2 Functional Genomics Facility, National Institute for Basic Biology, Okazaki, Japan; 3 Research Faculty of Agriculture, Hokkaido University, Sapporo, Japan; 4 The Graduate University for Advanced Studies (SOKENDAI), Hayama, Japan

**Keywords:** Arbuscular mycorrhiza, *Rhizophagus clarus*, de novo genome sequencing, Comparative genomics, Obligate symbiosis, Auxotrophy

## Abstract

**Background:**

Mycorrhizal symbiosis is one of the most fundamental types of mutualistic plant-microbe interaction. Among the many classes of mycorrhizae, the arbuscular mycorrhizae have the most general symbiotic style and the longest history. However, the genomes of arbuscular mycorrhizal (AM) fungi are not well characterized due to difficulties in cultivation and genetic analysis. In this study, we sequenced the genome of the AM fungus *Rhizophagus clarus* HR1, compared the sequence with the genome sequence of the model species *R. irregularis*, and checked for missing genes that encode enzymes in metabolic pathways related to their obligate biotrophy.

**Results:**

In the genome of *R. clarus*, we confirmed the absence of cytosolic fatty acid synthase (FAS)*,* whereas all mitochondrial FAS components were present. A KEGG pathway map identified the absence of genes encoding enzymes for several other metabolic pathways in the two AM fungi, including thiamine biosynthesis and the conversion of vitamin B6 derivatives. We also found that a large proportion of the genes encoding glucose-producing polysaccharide hydrolases, that are present even in ectomycorrhizal fungi, also appear to be absent in AM fungi.

**Conclusions:**

In this study, we found several new genes that are absent from the genomes of AM fungi in addition to the genes previously identified as missing. Missing genes for enzymes in primary metabolic pathways imply that AM fungi may have a higher dependency on host plants than other biotrophic fungi. These missing metabolic pathways provide a genetic basis to explore the physiological characteristics and auxotrophy of AM fungi.

**Electronic supplementary material:**

The online version of this article (10.1186/s12864-018-4853-0) contains supplementary material, which is available to authorized users.

## Background

The roots of most terrestrial plants in the world have a symbiotic relationship with filamentous fungi via mycorrhizae. Approximately 80% of land plants including 94% of Angiosperms form some type of association with mycorrhizae [1, 2]. Arbuscular mycorrhizae, a type of endomycorrhiza in which fungal hyphae enter the plant cells and shape highly branched structures named arbuscules, formed symbiotic relationships with land plants more than 400 million years ago [3]. In contrast, land plant associations with ectomycorrhizae, which is characterized by dense root-surrounding hyphae and intercellular hyphae between root cells, began about 190 million years ago [4]. Even in modern ecosystems, arbuscular mycorrhizae constitute the most abundant form of mycorrhizal association with angiosperms [2]. Thus, arbuscular mycorrhizal (AM) symbiosis is considered the most basic form of mycorrhizal symbiosis. Since many crops have associations with arbuscular mycorrhizae, AM symbiosis is also important agriculturally [5].

Fungi involved in AM symbioses belong to the fungal subphylum Glomeromycotina [6], which was formerly classified as phylum Glomeromycota [7]. AM fungi are obligate symbionts that cannot grow without their host plants, except in the very rare case of the cyanobacterial symbiont *Geosiphon pyriformis* [8, 9]. One reason for the obligate biotrophy of AM fungi is their dependence on hosts to supply a carbohydrate source [10]. Similar to ectomycorrhizal (ECM) fungi [11], AM fungi take up hexoses from the host [12, 13]. Recent studies have shown that AM fungi also import lipids from host plants [14, 15].

As obligate symbionts, AM fungi are very hard to culture in vitro, especially axenically [16]. This recalcitrance toward artificial culture prevents improvements derived from basic research as well as effective agricultural use. Therefore, the molecular genomics of AM fungi are not as advanced in comparison to other fungi. *Laccaria bicolor*, an ECM fungus, was the first mycorrhizal fungus to have its complete genome sequenced [17]. A reduction in the number of enzymes capable of degrading plant cell walls was hypothesized to result from the stable interaction with its host plant. To date, the genomes of multiple ECM fungi have been sequenced, including *Tuber melanosporum* (black truffle) and *Cenococcum geophilum* [18, 19]. The loss of plant cell wall-degrading enzymes (PCWDEs) has been confirmed to be a common feature of the genomes of ECM fungi as well as phytopathogenic fungi [20].

The first genome sequence of an AM fungus was published in 2013 for the model strain *Rhizophagus irregularis* DAOM197198 [21]. Analysis of that genome also indicated the loss of genes encoding PCWDEs. Genes for several fundamental metabolic enzymes such as thiamine synthase and type-I fatty acid synthase (FAS) were also absent [21, 22]. The recent discovery of lipid transport from plants to AM fungi is consistent with the inability of these fungi to synthesize fatty acids [14, 15]. Thus, discovery of missing genes for important biological processes may provide hints about the essential nutrient requirements of AM fungi. Such information is also useful for developing culture conditions for AM fungi.

The genome of *R. irregularis* was also sequenced for a study about heterokaryocity. *R. irregularis* DAOM197198 was found to contain homokaryotic nuclei with an intragenomic variant [23]. Several other strains of this fungal species have a dikaryon-like nuclear composition [24]. A strain of *R. clarus*, MUCL46238, was also sequenced to investigate effector-like secretory peptides [25]; however, there have been few comparative genomic studies of AM fungi, and the majority of conservative characteristics of AM fungi remain to be elucidated.

As for genes missing from AM fungi, Tang et al. [26] proposed that these genes be identified as “Missing Glomeromycotan Core Genes (MGCG)” in their transcriptome analysis of two *Gigaspora* species. Since transcriptome analysis is unable to detect genes with no or very low expression, the absence of MGCGs needs to be confirmed at the genomic level; however, the use of *Gigaspora* for genomic confirmation is problematic due to its extremely large genome size compared with other fungi [27].

To investigate a common genetic basis for obligate biotrophy in AM fungi at the whole-genome level, we de novo sequenced the genome of the HR1 strain of *R. clarus*. *R. clarus* is an AM fungal species belonging to the genus *Rhizophagus* and is characterized by producing larger spores than *R. irregularis*, *R. intraradices*, and several other *Rhizophagus* species [28]. *R. clarus* is a good candidate for agricultural investigations because a strain of *R. clarus* has been shown to have positive effects on soybeans and cotton [29]. We sequenced the HR1 strain of *R. clarus* and compared the sequence with that of *R. irregularis* in order to identify common missing metabolic pathways in AM fungi, including the previously proposed missing genes. This report details the gene composition of a fungal species representing the longest history of the mycorrhizal lifestyle.

## Results

### Genome sequencing and gene prediction

To investigate the gene repertoire of *R. clarus*, we sequenced genomic DNA from monoxenic cultures of the strain *R. clarus* HR1 by Illumina and PacBio sequencers. By k-mer analysis using Illumina reads, nuclei of *R. clarus* HR1 were found to be homokaryotic to the same degree as the model strain *R. irregularis* DAOM197198 (Additional file 1: Figure S1). The genome size of *R. clarus* was estimated to be approximately 146.4 Mbp, which is a little smaller than that of *R. irregularis* DAOM197198 (approximately 153 Mbp) [21] but much larger than the average fungal genome size (42.3 Mbp) [30]. Our assembly resulted in a total of 4424 scaffolds containing 116.41 Mbp sequences and an N50 length of 59.94 kbp (Table 1). Assessment of conserved gene comprehensiveness using BUSCO [31] revealed that 88.6% of the fungal conserved genes were complete genes. Similar to that of *R. irregularis* [21], the genome is AT-rich so that the GC content is as low as 27.2%. Since repeat masking following ab initio repeat modeling masked 36.04% of the total genome, the genome of *R. clarus* is up to 36% repeat-rich.

**Table 1:** Summary of genome assembly and gene prediction

Assembled genome		Predicted genes	
Assembly size	116.4 Mbp	Number of genes	27,753
Number of scaffolds	4424	Average of gene length	1465 bp
N50	59.94 kbp	Average of CDS length	1139 bp
GC %	27.2%	Average of protein length	379 aa
BUSCO genome benchmarks (fungi odb9)		BUSCO genome benchmarks (fungi odb9)	
Complete single copy	87.9% (257/290)	Complete single copy	94.8% (275/290)
Complete duplicated	0.7% (2/290)	Complete duplicated	2.1% (6/290)
Fragmented	3.4% (10/290)	Fragmented	0.7% (2/290)
Missing	8.0% (23/290)	Missing	2.4% (7/290)

Gene prediction combining ab initio prediction, protein mapping of *R. irregularis* proteins and transcript mapping resulted in the identification of 27,753 coding genes (Table 1). BUSCO assessment detected 96.9% of the conserved genes as complete sequences, indicating that this gene catalogue is highly comprehensive. The total number of genes found for *R. clarus* is similar to that of *R. irregularis* (28,232 genes reported in Tisserant et al. 2013 [21]). This number is smaller than that reported in a recent study of *R. irregularis* (41,572 genes), but similar to the reported number of reliable genes (27,860 genes) [32]. Since most fungi have fewer than 20,000 coding genes [30], harboring many genes may be a characteristic of AM fungi.

### Absence of cytosolic fatty acid synthase

**Fig. 1:**
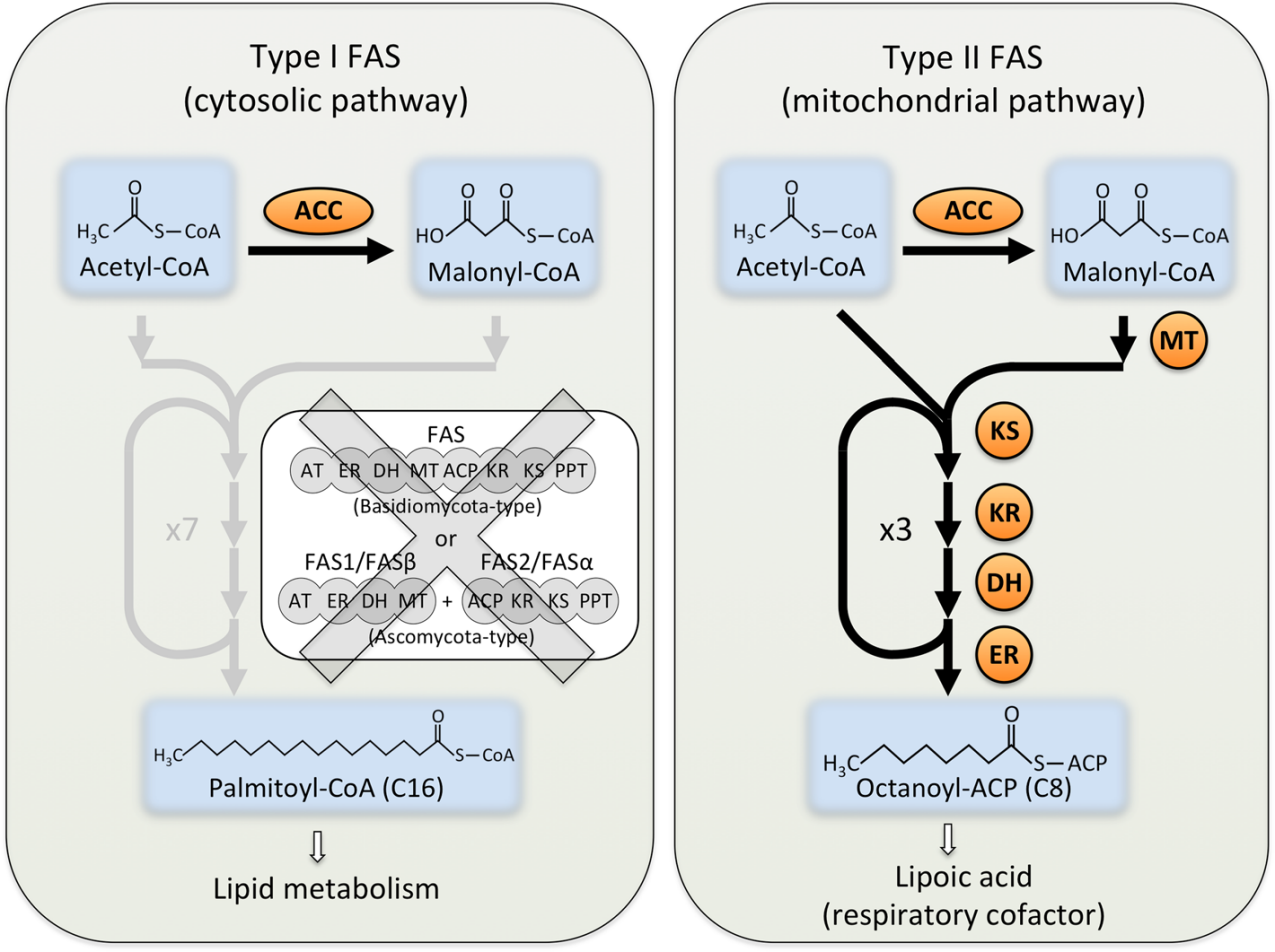
Fatty acid biosynthetic pathways in the genome of AM fungi. Black arrows and genes with orange backgrounds represent pathways and enzymes present in two AM fungi (*R. clarus* and *R. irregularis*). Gray arrows and genes represent absent pathways and enzymes in AM fungi. Abbreviations are as follows: ACC for acetyl-CoA carboxylase, MT for malonyl-CoA ACP transacylase, KS for 3-ketoacyl synthase, KR for 3-ketoacyl reductase, DH for enoyl dehydratase, ER for enoyl reductase

Being an obligate symbiont, AM fungi presumably rely on some important biological processes supplied by their host plants. Recently, fatty acids were identified as important factors for auxotrophy of AM fungi [33–35]. In general, fungi and animals have two FAS gene sets: type I FAS and type II FAS (Fig. 1) [36, 37]. The type I FAS consists of a cytosolic gene or genes with multiple domains that produce long chain fatty acids. Type I FAS is an octa-functional single gene in animals and most basidiomycota such as *Laccaria* and *Ustilago*; however, two tetra-functional type I FAS genes, FAS1/FASβ and FAS2/FASα, are found in ascomycota such as *Saccharomyces* and *Aspergillus* [38]. In contrast, type II FAS genes are bacterial-like gene sets used in mitochondria and consisting of individual subunit genes. Whereas type I FAS produces long chain (C16) fatty acids, type II FAS synthesizes the mitochondrial respiratory cofactor lipoic acid [39]. In the genome of *R. irregularis*, Wewer et al. [22] reported the absence of type I FAS genes, whereas most of the type II FAS genes were present. On the other hand, Vijayakumar et al. [40] claimed that AM fungi could synthesize FA as demonstrated by expression analysis of FAS-related genes.

To determine at the genomic level whether the missing FAS genes are a common feature of AM fungi, we surveyed genes homologous to type I and type II FAS genes in the genome sequences and predicted genes of *R. clarus* as well as in the improved gene catalogue for *R. irregularis*. In our homology search, all components of type II FAS genes including enoyl dehydratase, which had not been discovered in *R. irregularis* prior to the previous report [22], were found in both *R. clarus* and *R. irregularis* (Additional file 2: Table S1, Fig. 1). Thus, these AM fungi have a complete set of genes encoding enzymes for the type II FAS pathway. In contrast, a homology search for type I FAS genes in the *R. clarus* genome only identified genes that are too short to correspond with multifunctional FAS genes (Additional file 2: Table S1). Given their high similarity to the type II FAS component, these FAS-like genes are hypothesized to be more similar to type II FAS genes than type I FAS genes. An *R. irregularis* gene EXX52120.1, annotated as a “tetra-functional FAS subunit” by Lin et al. [23] and used in Vijayakumar et al. [40], was also confirmed to encode a malonyl-CoA ACP transacylase component of a type II FAS (Additional file 2: Table S1). Therefore, these AM fungi lack specific genes for the cytosolic FAS pathway, which plays a pivotal role in the biosynthesis of long-chain fatty acids.

### Common missing pathways for vitamins and cofactors

Three previously published reports identified FAS genes, thiamine biosynthesis genes and MGCG as missing from AM fungi [21, 22, 26]. Thus, we checked for the absence of MGCG in our *R. clarus* genomic DNA dataset. Among 39 MGCGs, eight genes involved in thiamine biosynthesis were all absent from the *R. clarus* and *R. irregularis* genomes, suggesting common loss of genes for thiamine synthesis in AM fungi (Additional file 3: Table S2). By contrast, three genes shown to be present in *R. irregularis* in a previous study, AAD15, PHO89, and URE2, were present in both *R. irregularis* and *R. clarus*, indicating that the absence of these three genes is not common among AM fungi (Additional file 3: Table S2). Moreover, homologs of YHB1 and RHR2, also identified as MGCG in Tang et al. [26], were also discovered in two *Rhizophagus* species with comparatively low scores, suggesting that these genes may also be present in *Rhizophagus* species.

In order to find novel missing genes, we next searched for indications of missing metabolic pathways in *R. clarus and R. irregularis* using the KEGG metabolic pathway mapper [41]. When compared to the well-annotated genomes of two saprotrophic fungi, *Saccharomyces cerevisiae* and *Aspergillus oryzae*, the absence of genes encoding enzymes in the FAS and thiamine biosynthetic pathways in the *R. clarus* and *R. irregularis* genomes was confirmed again (Additional file 1: Figure S2, Additional file 3: Table S2). In addition, several other enzymes were also found to be absent from both AM fungi. For example, in the metabolic pathway for vitamin B6, AM fungi lack genes encoding enzymes that convert pyridoxal 5′-phosphate (the active form of vitamin B6) or pyridoxal into related derivatives such as pyridoxine and pyridoxamine, in contrast to the presence of these genes in *Saccharomyces* and *Aspergillus* (Additional file 1: Figures. S2 and S3, Additional file 4: Table S3). Although AM fungi can synthesize bioactive forms of vitamin B6, the lack of derivatives might affect some metabolic processes. These specific losses of vitamin metabolisms were unique characteristics among fungi (Additional file 5: Table S4).

### Carbohydrate availability

**Fig. 2:**
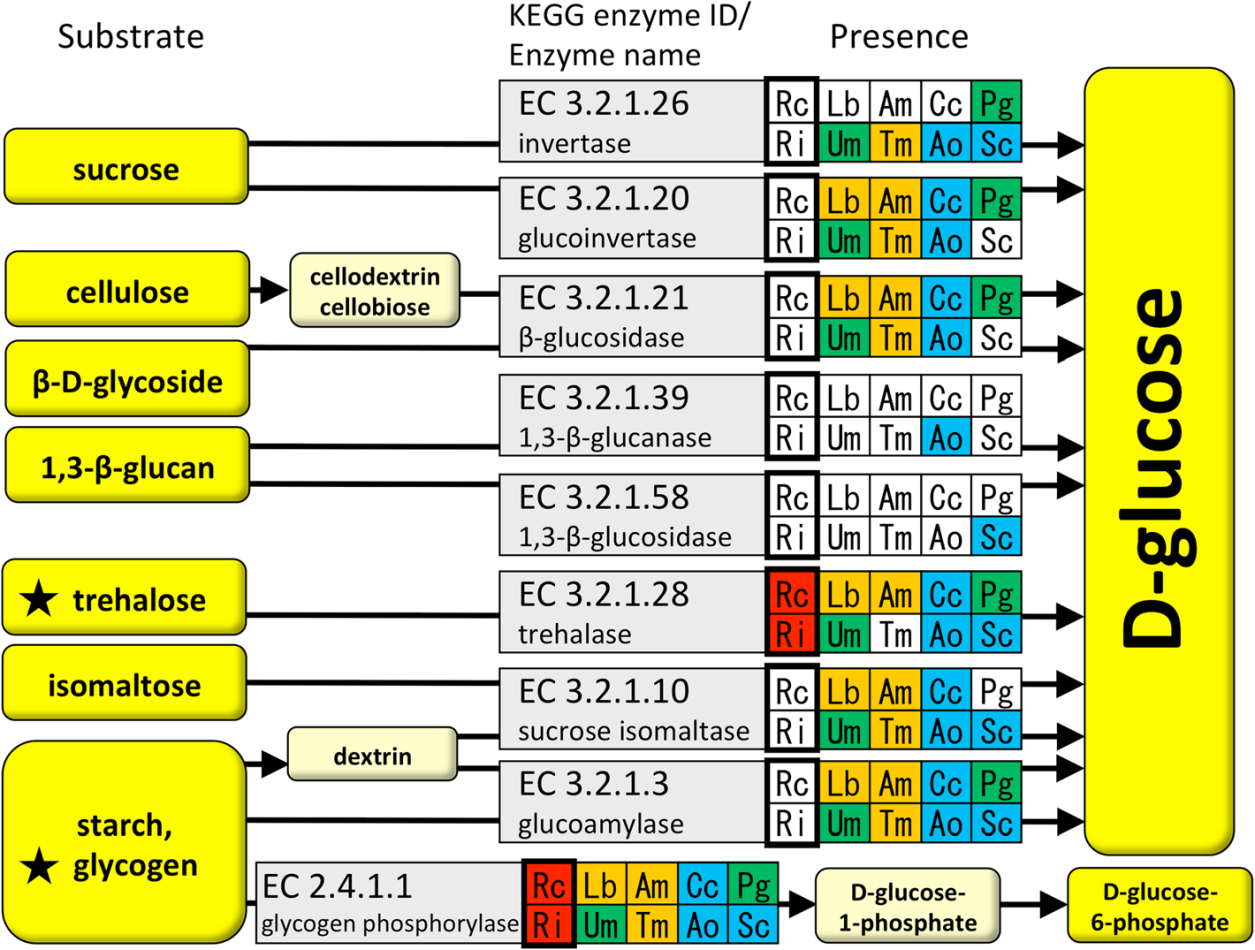
Glucose-producing hydrolases of AM fungi and other fungi. Enzymes present in organisms are indicated by colored backgrounds. Red, orange, green and blue indicate AM, ECM, pathogenic and saprotrophic fungi, respectively. Rc, *R. clarus*; Ri, *R. irregularis*; Lb, *Laccaria bicolor*; Am, *Amanita muscaria*; Cc, *Coprinopsis cinerea*; Pg, *Puccinia graminis*; Um, *Ustilago maydis*; Tm, *Tuber melanosporum*; Ao, *Aspergillus oryzae*; Sc, *Saccharomyces cerevisiae*. Stars indicate polysaccharides that AM fungi can synthesize

Generally, mycorrhizal fungi are known to have fewer PCWDEs due to their close symbiotic relationship with plants [20, 21]. This loss of polysaccharide hydrolases may also be related to the amount of sugars supplied from host plants. Therefore, we closely examined the carbohydrate-degrading processes of *R. clarus* and *R. irregularis* using the KEGG metabolic pathway mapper. We found that the putative genes for many enzymes involved in glucose production by polysaccharide hydrolysis, including sucrose glucohydrolases (invertase EC3.2.1.26 and glucoinvertase EC3.2.1.20, from sucrose to glucose), β-glucosidase (EC3.2.1.21, from cellulose-derived cellobiose to glucose), glucoamylase (EC3.2.1.3, from starch and glycogen to glucose) and sucrose-isomaltase (EC3.2.1.10, from dextrin to glucose), were absent from *R. clarus* and *R. irregularis* (Fig. 2, Additional file 5: Table S4). The absence of invertase and the deficiency in sucrose availability are consistent with previous reports on *R. irregularis* [21, 26]. Our findings also identified the loss of genes encoding other glucose-producing hydrolases such as β-glucosidase, which is present in most other fungi including ECM and plant-pathogenic fungi (Fig. 2, Additional file 5: Table S4). A previous study reported that several biotrophic fungi such as *Amanita muscaria* and *Ustilago maydis* lack genes encoding cellulose-binding proteins, which function in cellulose degradation [20]. Our result suggests that AM fungi may have a reduced ability to utilize cellulose as a substrate compared to other fungi because the absence of genes encoding β-glucosidases is specific to AM fungi. We also found that a gene encoding a glycogen-degrading enzyme, glucoamylase (EC 3.2.1.3), is also absent specifically from AM fungi, suggesting low glycolysis activity in AM fungi.

In our search, genes encoding trehalase (EC 3.2.1.28, from trehalose to glucose) and glycogen phosphorylase (EC 2.4.1.1, from glycogen to glucose-1-phosphate) were the only carbohydrate hydrolase genes capable of producing glucose-related hexoses in AM fungi (Fig. 2, Additional file 5: Table S4). The presence of trehalase is consistent with the results of two previous reports [42, 43]. Since AM fungi synthesize trehalose and glycogen [42, 44, 45], these putative enzymes are hypothesized to hydrolyze fungal-produced products.

These results suggest that AM fungi are unable to utilize polysaccharides, such as cellulose and other glucans, that are found in their environment, whereas ECM fungi partly have the ability to use these carbohydrate sources. Thus, AM fungi may be more dependent on plants for acquiring carbohydrates than ECM fungi.

### Comparison of two AM fungal species related to metabolic pathways

Since *R. clarus* HR1 was originally isolated from acidic soil and has an optimal effect on plants growing under low pH conditions [46], we checked pathways related to acid tolerance. In the case of algae, convergent loss of fermentation pathways producing organic acids such as lactate, formate and acetate causing cytosolic acidification in acidophilic species has been reported [47]. We investigated whether these pathways exist in two *Rhizophagus* species, and consequently found that *R. clarus* HR1 retains the same repertoire of those enzymes to that of *R. irregularis* (Additional file 1: Figure S4). Similarly, we could not find other significant differences from the genome-wide comparison of metabolic pathways between *R. irregularis* and *R. clarus*. Thus, morphological and physiological differences of these two AM fungal species may depend on other regulatory mechanisms different from unique metabolic processes.

## Discussion

### Genomic features of *R. clarus* HR1

*R. clarus* HR1 has an AT-rich nuclear genome of approximately 146Mbp (Table 1), which is similar to that of the model strain *R. irregularis* DAOM197198 [21]. This result suggests that approximately 150 Mbp is the standard genome size for the genus *Rhizophagus*.

The model AM fungal species *R. irregularis* has strains with haploid-type and diploid-type karyotypes [24]; the model strain DAOM197198 is a haploid-type strain [23]. In this study, we determined that *R. clarus* HR1 has haploid-type homokaryotic nuclei since the k-mer distribution pattern of *R. clarus* HR1 is similar to that of *R. irregularis* DAOM197198. So far, diploid-type strains of *R. clarus* have not been discovered, and the genome heterokaryocity of AM fungi other than *R. irregularis* largely remains to be elucidated.

### Energy metabolism of AM fungi

Since AM fungi are biotrophic organisms, they rely on host plants to serve as energy sources. AM fungi are known to import sugars in the form of hexoses [13], similar to how ECM fungi acquire sugars [11]. The reduced number of carbohydrate-reactive enzymes in mycorrhizal fungi also suggests that sugars are acquired from plants [20]. In this study, we revealed the extreme absence of genes encoding glucose-producing oligosaccharide hydrolases in AM fungi, whereas the genomes of ECM fungi have genes to encode most of these enzymes (Fig. 2). Thus, AM fungi are hypothesized to have completely abolished glucose acquisition except for what is imported from their host plants. This absolute host-dependency of AM fungi may be reflective of its long symbiotic history. Extraordinarily large spores may also correlate with the loss of independent sugar acquisition because germinating AM fungi must depend on their own energy sources before a mycorrhizal symbiosis can be established.

AM fungi are also known to accumulate lipids that comprise 46 ~ 70% of the spore weight [48]. These high lipid contents are likely stored for germination and initial growth before successful infection. The ability of AM fungi to synthesize lipids is sometimes debated. The existence of fatty acid species specific to AM fungi has been known for a long time [48]. AM fungi are unable to produce long chain (C16) fatty acids without host plants, although they can elongate C16 fatty acids into fungal-specific fatty acid species [49]. At the genomic level, Tisserant et al. [50] found transcripts homologous to FASα and FASβ, but Wewer et al. [22] identified those genes as type II FAS subunits and reported the absence of type I FAS genes in the *R. irregularis* genome. By contrast, Vijayakumar et al. [40] claimed that AM fungi could synthesize lipids based on the expression and localization analysis of CEM1 (a type II ketoacyl synthase) and a gene annotated as “tetra-functional fatty acid synthase subunit FAS1” (EXX52120.1). In our study, we identified the putative “FAS1” as a subunit of a type II FAS and confirmed the absence of type I FAS genes from the genomes of both *R. clarus* and *R. irregularis* (Additional file 2: Table S1). Though type II FAS enzymes can synthesize long-chain fatty acids in vitro [51], the ability to synthesize long-chain fatty acids in vivo is doubtful since type I FAS and type II FAS are non-redundant and are unable to complement each other unless their localization patterns are altered [52–54]. Observations of fatty acid importation from host plants [14, 15] and mycorrhizal defects caused by lipid-related plant mutants [55, 56] also support the hypothesis that AM fungi rely on their hosts for fatty acids. Thus, AM fungi are hypothesized to lack the ability to synthesize long-chain fatty acids.

Although AM fungi accumulate lipids, many ECM and saprotrophic fungi store carbohydrates such as glycogen, trehalose and mannitol as their carbon sources [57–59]. A possible reason for this difference may be because AM fungi have to store lipids that are essential for membrane construction in their cells. These differences in primary storage compounds may also be correlated with the availability of polysaccharides. AM fungi have lost the gene for glucoamylase, a glycolytic enzyme that is highly conserved in other fungi (Fig. 2). Thus, AM fungi may not be able to use glycogen effectively compared to other fungi; thus, glycogen is less important as a carbon source.

### AM fungi and difficulties with cultivation

AM fungi are known to be very difficult to cultivate axenically [16]. The reason for this difficulty is thought to be their auxotrophic dependency on symbiosis. The growth of AM fungi is hypothesized to depend on many other compounds in addition to lipids and carbohydrates. We confirmed the absence of several metabolic pathways associated with the biosynthesis of vitamin B6 derivatives and thiamine synthesis (Additional file 1: Figure S2, Additional file 5: Table S4). Missing of thiamine synthetic pathway, which is supposed to be essential for infection in rust fungi [60], suggests that AM fungi may have non-thiamine dependent infection system. Since these vitamins are essential for events other than infection, addition of these compounds to media may improve the culture efficiency of AM fungi.

In addition to conserved characteristics, AM fungi also have species-specific characteristics for successful culture. The effectiveness of symbiotic fungi on plant growth also differs by species and strains [61]. Though our analysis could not find genetic difference of AM fungal species contributing physiological characteristics yet, the draft genomic sequence data for *R. clarus* HR1 may help to understand how its genetic background influences acid tolerance and promotes effective plant growth.

## Conclusions

In this study, we sequenced the genome of an AM fungus, *R. clarus* HR1, and compared the data with that from a model AM species, *R. irregularis* DAOM197198. We confirmed that genes for several metabolic pathways such as cytosolic fatty acid biosynthesis and thiamine biosynthesis were absent in both AM species. We also found that metabolic genes such as those encoding enzymes that synthesize vitamin B6 derivatives were commonly absent. As for sugar metabolism, AM fungi lack almost all the genes encoding polysaccharide hydrolases that produce glucose except trehalase and glycogen phosphorylase, whereas most of these polysaccharide hydrolase genes are present in other phyto-biotrophic fungi. These findings support observations of the high host dependency of AM fungi.

## Methods

### Sample preparation

The HR1 fungal strain of *R. clarus* was isolated from Hazu, Nishio, Aichi, Japan [46]. This strain is available from the MAFF Gene Bank as MAFF520076. Fungal samples were cultured monoxenically with *Agrobacterium rhizogenes*-induced hairy roots of carrot (*Daucus carota*) [62] growing on M medium [63]. After 2 months incubation, roots of the host plant were removed and fungal tissues were collected using citrate-mediated lysis of the culture medium [64].

### DNA extraction and amplification

Genomic DNA was extracted by a modified CTAB method as described in Maeda et al. [32]. Extracted DNA was purified with gravity-flow, anion-exchange tips (Genomic-Tip 20/G, Qiagen, Netherlands).

For PacBio sequencing, genomic DNA was amplified using a REPLI-g Single Cell Kit (Qiagen, Netherlands) after selection of long DNA (> 6 kbp) using the BluePippin DNA size-selection system (Sage Science, USA). To reduce artificial effects resulting from amplification, extracted gDNA was separated into six tubes before amplification, and each sample was amplified independently. After amplifying the entire genome, DNA was purified again with Genomic-Tips 20/G.

### Sequencing

#### Illumina sequencing

Paired-end libraries of two different insertion sizes (180 bp and 600 bp) were constructed for Illumina sequencing using approximately 1 μg of genomic DNA. Libraries were prepared according to the standard Illumina TruSeq DNA protocol. Libraries were sequenced by using HiSeq 1500 for sequencing reads of 126 bp each. The total number of sequencing reads obtained from each genome was approximately 18.7 Gbp (74 Mreads) for PE180 and 33.3 Gbp (123 Mreads) for PE600.

#### PacBio sequencing

Approximately 10 μg each of amplified gDNA was used for PacBio library construction. Sequencing libraries were constructed following the manufacturer’s protocol (Pacific Bioscience, USA). Libraries were sequenced with a PacBio RS II instrument and resulted in 26.0 Gbp of total sequencing reads.

### Genome assembly

Sequence reads were trimmed using cutadapt 1.8.1 [65]. Sequencing adapters and low-quality bases at the 5′-ends (7 bp for Illumina PE180, 10 bp for Illumina PE600 and 100 bp for PacBio reads) and the 3′-ends (QV < 20 region for Illumina reads) were removed. After trimming, sequencing errors in the PacBio reads were removed using Sprai 0.9.9.20 [66]. To diminish amplification-based errors, the script “ezez4makefile_v4.pl” in the Sprai 0.9.9.20 package was used with the “filter_same_lib” option.

Illumina reads were first used for k-mer analysis using Jellyfish [67]. Using karyotype information and the preliminary estimated genome size, assembly of Illumina reads and estimation of the precise genome size were performed with AllPaths-LG 44837 [68]. The initial “estimated genome size” parameter was set to 150,000,000. The resulting contigs were scaffolded with Opera-LG 2.0.6 using Illumina reads and PacBio error-corrected reads [69]. Gaps in the scaffolds were filled with PBJelly using the PBSuite 15.8.24 package with PacBio error-corrected reads using parameters identified by the manufacturer [70]. Sequential errors were corrected with Pilon 1.22 using Illumina reads [71] after mapping the reads with bowtie2 2.2.0 [72].

To remove contaminated sequences, scaffolds were subjected to a homology search of the NCBI NR database using GhostZ 1.0.0 [73]. In the search results, hits to *Anthurium amnicola* sequences were removed since sequences annotated to this higher plant in the NR database were thought to contain contaminating fungal sequences. Using homology search results from GhostZ, the origins of the scaffolds were predicted with MEGAN 6.6.7 [74]; scaffolds without fungal origins were discarded. Short scaffolds (< 1 kbp) were also removed. The completeness of the genome was assessed using BUSCO 2.0 [31] with the gene set “Fungi odb9” and the augustus gene model of *Rhizopus oryzae*.

### Masking of repetitive sequences

Repeat sequences were identified with RepeatMasker 4.0.6 and RepeatModeler 1.0.8 [75]. Repeat motifs were constructed de novo with RepeatModeler, and then the repetitive regions of the draft genome were masked with RepeatMasker. Parameters for RepeatModeler and RepeatMasker were not changed from the default values.

### Gene prediction

For evidence-based gene prediction, RNA-seq was performed with the Illumina system. Total RNA was extracted with an RNeasy Plant Mini Kit (Qiagen, Netherlands). An RNA-seq library was constructed from 200 ng of RNA using the TruSeq Stranded RNA Library Prep Kit (Illumina, USA). After quality evaluation, the library was sequenced by an Illumina HiSeq 1500.

RNA-seq reads were trimmed with cutadapt to remove adapter sequences and low-quality bases (5′-ends 15 bp and 3′-ends QV score < 30). Trimmed reads were mapped to genomic data with Tophat 2.1.1 [76]. Using the Tophat result, genome-guided transcript assembly was achieved with Trinity 2.0.6 [77]. The resulting transcripts were encoded by 29,997 genes; the total number of identified sequences including splice variants were up to 39,663 sequences. The total number of identified bases was approximately 42.53 Mbp and the N50 was 1653 bp. ORFs were also predicted with TransDecoder 3.0.1 [78] and 30,826 ORFs including 15,941 complete ORFs were predicted.

Since several conserved genes were found in masked regions, gene prediction was performed using unmasked genomic data. Gene predictions were accomplished with four different methods: GeneMark-ES [79], Augustus [80], Exonerate protein mapping [81], and Exonerate transcript mapping [82]. A totally ab initio prediction was performed using GeneMark-ES 4.33 with default parameters and resulted in 27,995 genes. Gene prediction with taxonomy-based parameters and transcript-based hints was conducted using Augustus 3.1.0 with the gene model of *R. irregularis* [32] and a hint-file prepared from BLAT mapping of the transcripts [83]. Augustus predicted 28,576 genes. Gene regions were also predicted by mapping protein sequences of the related species *R. irregularis* [32] using Exonerate 2.2.0 with the “--percent” option set as 80. This mapping resulted in 9994 mapped loci. ORFs from transcripts were also mapped with Exonerate with the “--percent” option set as 90 and resulted in 16,723 loci. Each result was integrated into the final gene models with EVidenceModeler 1.1.1. Weights for each result were set as follows: Genemark-ES: Augustus: Exonerate *R. irregularis* protein mapping: Exonerate transcript mapping = 2: 1: 3: 3.

### Investigation of missing genes and metabolic pathways

FAS genes and MGCGs were identified by BLAST searches using deduced protein sequences of *S. cerevisiae* as queries. For the FAS genes, the corresponding sequences of *Aspergillus nidulans*, *Laccaria bicolor*, *Puccinia graminis* and *Ustilago maydis* were also used as BLAST queries. A BLAST result with an e-value < 10^− 5^ was regarded as a positive hit.

Metabolic pathways were searched using the KEGG metabolic pathway map [41]. KEGG pathways were annotated from predicted gene catalogues using GhostKoala with “genus_prokaryotes + family_eukaryotes” databases at the KEGG web server [84]. Annotated KEGG lists were also mapped at the KEGG website.

### Reference sequences

Draft genomes from two previous studies [21, 23] were downloaded from the websites reported in their published papers. Gene lists and sequence reads of *R. irregularis* DAOM197198 were also used from Maeda et al. [32]. Gene repertory files of other fungi used as references were downloaded from JGI MycoCosm [85, 86] or NCBI Genome [87]. Genomic information of *Laccaria bicolor* (v2.0) [17], *Amanita muscaria* Koide (v1.0) [20], *Coprinopsis cinerea* Okayama-7 [88], *Ustilago maydis* 521 (v2.0) [89], *Puccinia graminis* f. sp. Tritici (v2.0) [90], *Tuber melanosporum* Mel28 (v1.0) [18], *Neurospora crassa* [91], *Saccharomyces cerevisiae* S288C [92], *Aspergillus oryzae* RIB40 [93, 94], *Aspergillus nidulans* [94, 95], *Taphrina deformans* [96] and *Shizosaccharomyces pombe* [97] were downloaded from JGI website. Gene catalog of *Suillus luteus* (GCA_000827255.1) [20], *Rhizoctonia solani* (GCA_000524645.1) [98], *Auricularia subglabra* (GCF_000265015.1) [99], *Cryptococcus neoformans* (GCF_000149245.1) [100], *Magnaporthe oryzae* (GCF_000002495.2) [101], *Fusarium oxysporum* (GCF_000149955.1) [102] and *Cenococcum geophilum* (GCA_001692895.1) [19] were downloaded from NCBI website.

## Additional files

Additional file 1 : **Figure S1**. The k-mer (k = 31) content of *R. clarus* (left panel) and *R. irregularis* (right panel) obtained from HiSeq short-reads analyzed with Jellyfish [65]. **Figure S2**. Common missing pathways in two *Rhizophagus* species. **Figure S3**. Pathways in vitamin B6 metabolism. **Figure S4**. Fermentation pathways converting pyrvate into lactate, formate and acetate, which causes cytosolic acidification. (DOCX 1385 kb)

Additional file 2 : **Table S1**. Homology search results for FAS genes. (XLSX 51 kb)

Additional file 3 : **Table S2**. MGCGs in *R. clarus* compared with other AM fungi. (XLSX 36 kb)

Additional file 4 : **Table S3**. KEGG pathways affected by genes missing in *R. clarus* and *R. irregularis* but present in other fungi. (XLSX 34 kb)

Additional file 5 : **Table S4**. The presence of notable KEGG metabolic enzymes in 20 fungal species assessed by GhostKOALA. (XLSX 24 kb)

## Abbreviations

AM: Arbuscular mycorrhizal
ECM: Ectomycorrhizal
FAS: Fatty acid synthase
MGCG: Missing Glomeromycotan core genes
PCWDE: Plant cell wall-degrading enzymes
