# Supplementary material for: The genome of Rhizophagus clarus HR1 reveals a common genetic basis for auxotrophy among arbuscular mycorrhizal fungi
Source: BMC Genomics. 2018 Jun 18;19:465. doi: 10.1186/s12864-018-4853-0 (PMC6007072; doi:10.1186/s12864-018-4853-0)
Supplement: Supplementary file 1 — : Figure S1. The k-mer (k = 31) content of R. clarus (left panel) and R. irregularis (right panel) obtained from HiSeq short-reads analyzed with Jellyfish [65]. Figure S2. Common missing pathways in two Rhizophagus species. Figure S3. Pathways in vitamin B6 metabolism. Figure S4. Fermentation pathways converting pyrvate into lactate, formate and acetate, which causes cytosolic acidification. (DOCX 1385 kb) [file 12864_2018_4853_MOESM1_ESM.docx]

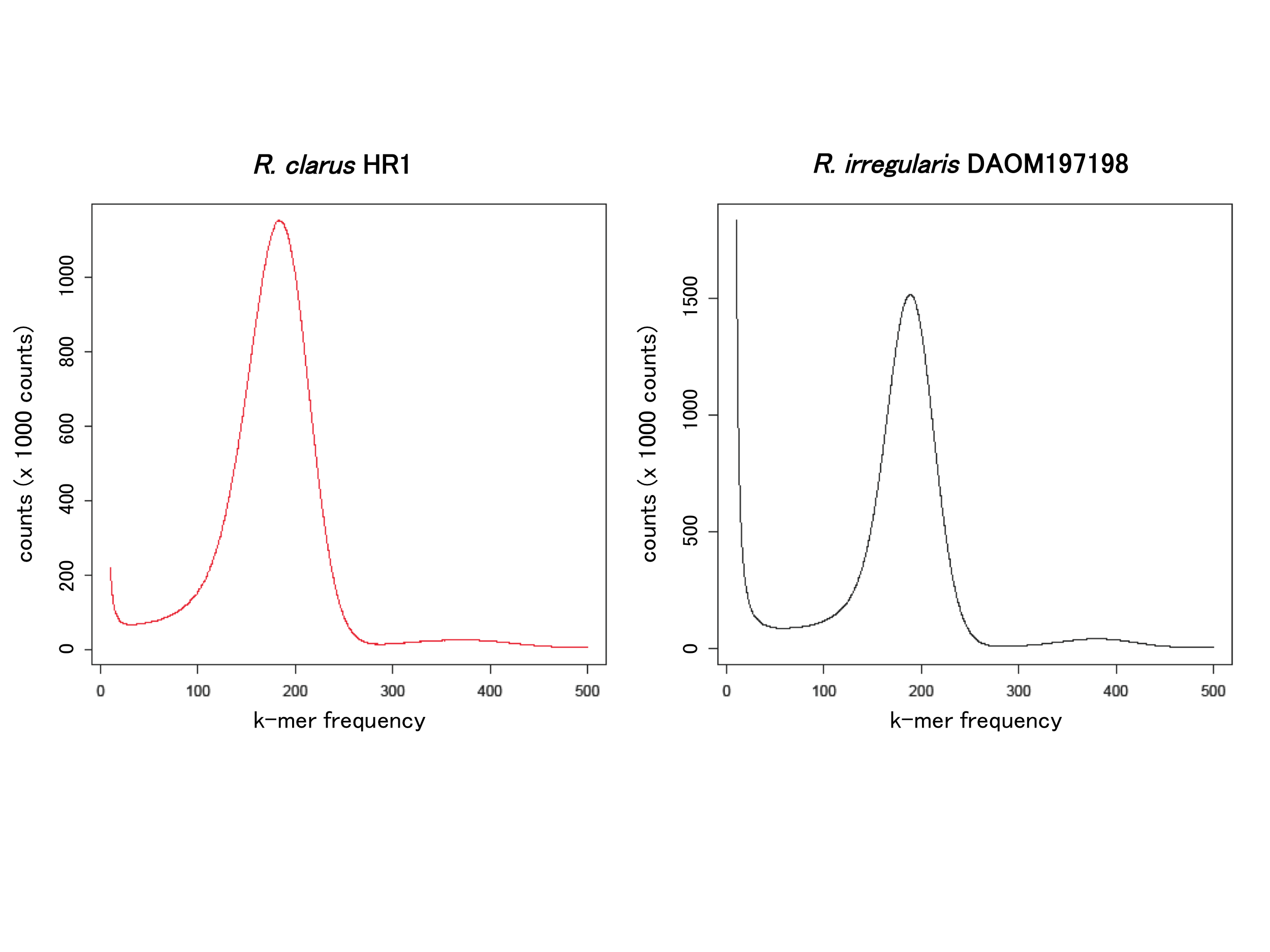


Figure S1 The k-mer (k=31) content of *R. clarus* (left panel) and *R. irregularis* (right panel) obtained from HiSeq short-reads analyzed with Jellyfish [65].


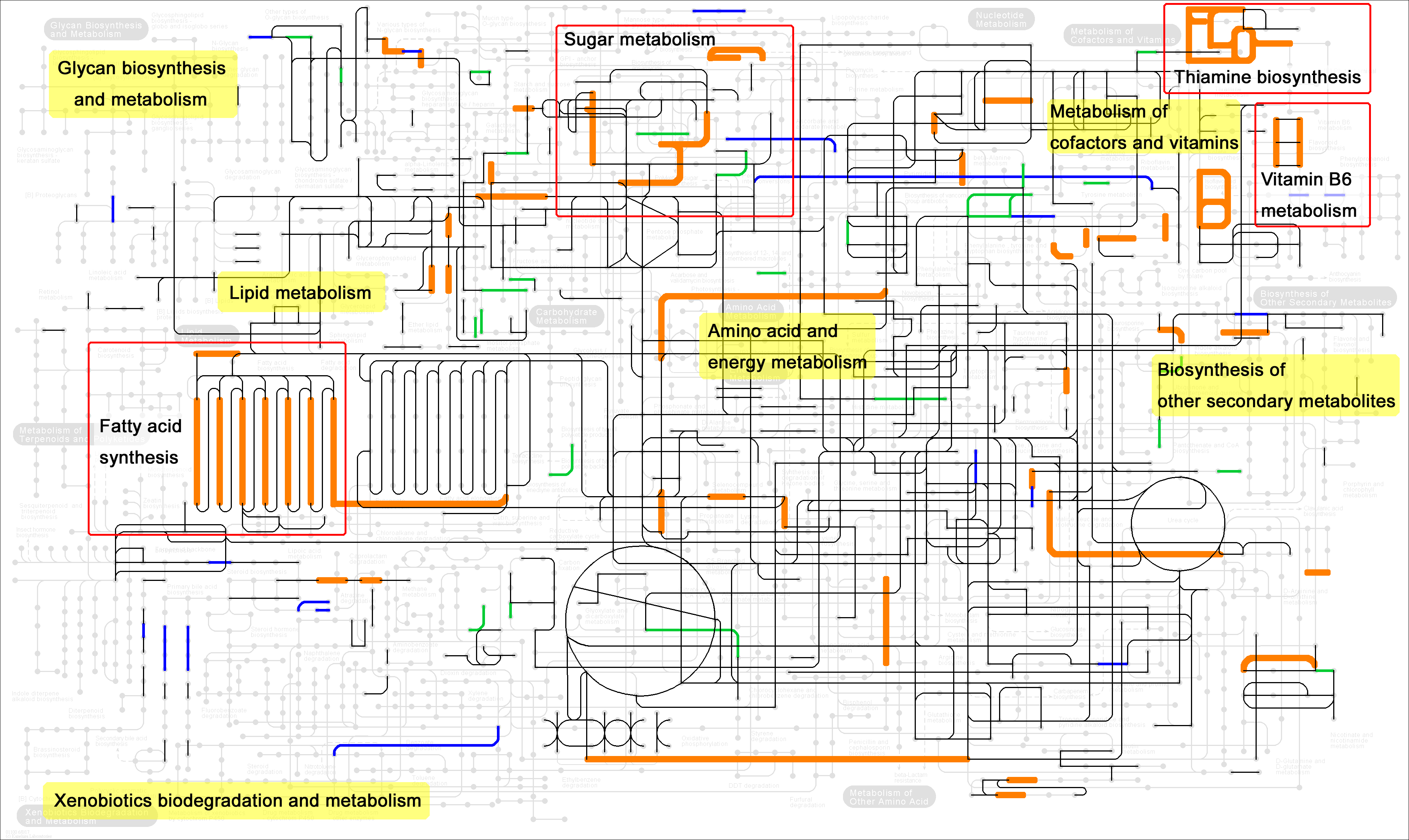


Figure S2 Common missing pathways in two *Rhizophagus* species. The thick orange lines indicate enzymatic pathways whose genes are absent in *R. clarus* and *R. irregularis* but present in *Saccharomyces cerevisiae* and *Aspergillus oryzae*. Black lines indicate enzymatic pathways whose genes are present in *R. clarus* and *R. irregularis*. Green and blue lines indicate enzymatic pathways whose genes are present in either *R. clarus* or *R. irregularis*, respectively.


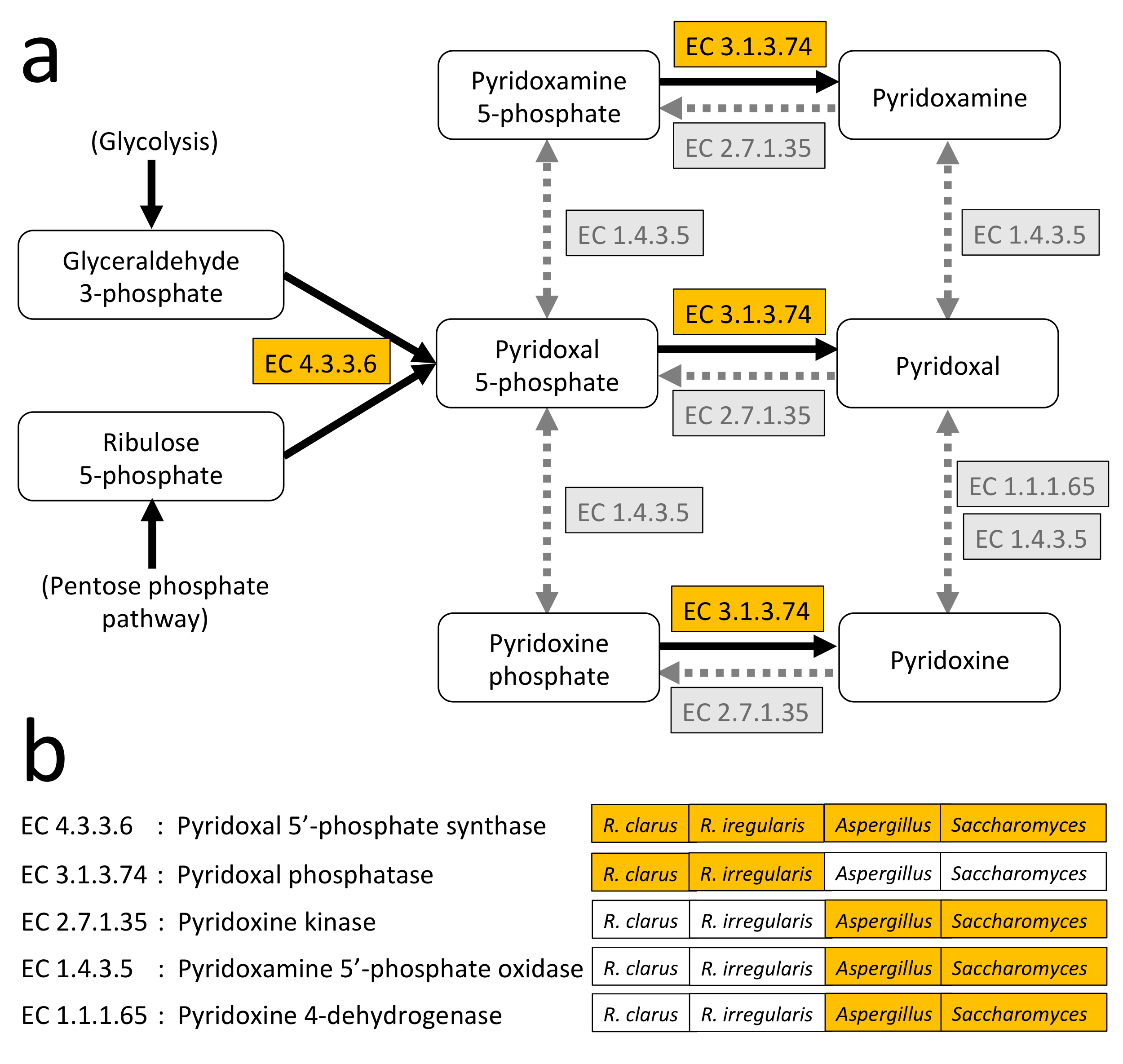


Figure S3 Pathways in vitamin B6 metabolism. **a** Metabolic pathway maps. KEGG numbers shown with an orange background indicate pathways for which encoding genes are present in AM species. **b** Distribution of deduced enzymes in four fungal species. If genes encoding the indicated enzyme are present in each species, the species name is shown with an orange background.


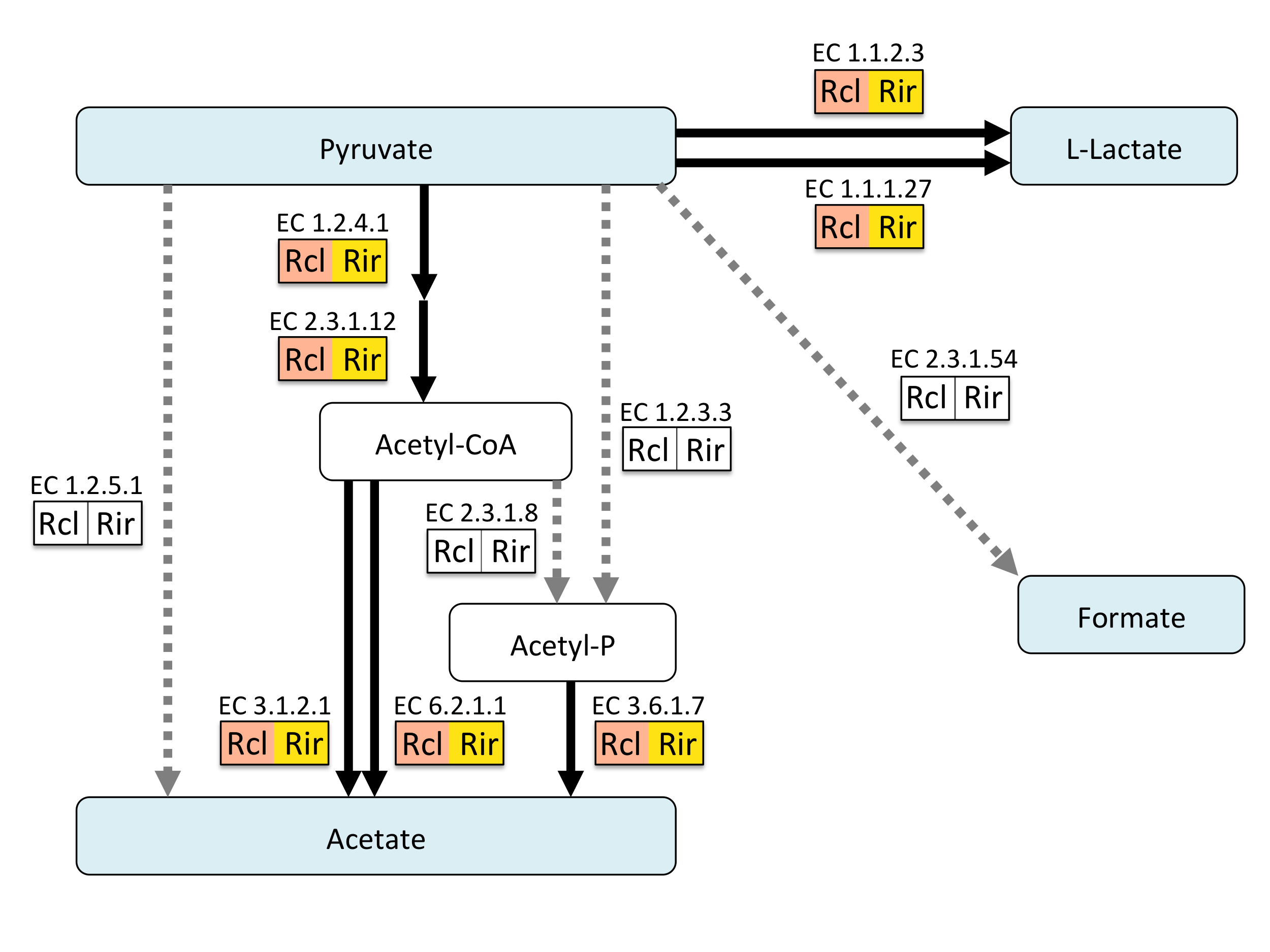


Figure S4 Fermentation pathways converting pyrvate into lactate, formate and acetate, which causes cytosolic acidification. The enzymes present in each AM fungus are colored.
